# Material Classification and Aging Time Prediction of Structural Metals Using Laser-Induced Breakdown Spectroscopy Combined with Probabilistic Neural Network

**DOI:** 10.3390/ma16165599

**Published:** 2023-08-12

**Authors:** Qian Wang, Guowen Li, Yuhua Hang, Silei Chen, Yan Qiu, Wanmeng Zhao

**Affiliations:** 1 School of Sciences, Xi’an University of Technology, Xi’an 710048, China; qianwang@xaut.edu.cn (Q.W.); 2210920069@stu.xaut.edu.cn (G.L.); 2220920070@stu.xaut.edu.cn (W.Z.); 2 Suzhou Nuclear Power Research Institute Co., Ltd., Suzhou 215004, China; hangyuhua@cgnpc.com.cn; 3 Key Laboratory of Physical Electronics and Devices, Ministry of Education, Faculty of Electronic and Information Engineering, Xi’an Jiaotong University, Xi’an 710049, China

**Keywords:** laser-induced breakdown spectroscopy, probabilistic neural network, structural metal, aging, machine learning

## Abstract

In this paper, laser-induced breakdown spectroscopy (LIBS) combined with a probabilistic neural network (PNN) was applied to classify engineering structural metal samples (valve stem, welding material, and base metal). Additionally, utilizing data from the plasma emission spectrum generated by laser ablation of samples with different aging times, an aging time prediction model based on a firefly optimized probabilistic neural network (FA-PNN) was established, which can effectively evaluate the service performance of structural materials. The problem of insufficient features obtained by principal component analysis (PCA) for predicting the aging time of materials is addressed by the proposal of a time-frequency feature extraction method based on short-time Fourier transform (STFT). The classification accuracy (ACC) of time-frequency features and principal component features was compared under PNN. The results indicate that, in comparison to the PCA feature extraction approach, the time-frequency feature extraction method based on STFT demonstrates higher accuracy in predicting the time of aging materials. Then, the relationship between classification accuracy (ACC) and settings of PNN was discussed. The ACC of the PNN model for both the material classification test set and the aging time test set achieved 100% with Firefly (FA) optimization algorithms. This result was also compared with the ACC of ANN, KNN, PLS-DA, and SIMCA for the aging time test set (95%, 87.5%, 85%, and 62.5%, respectively). The experimental results demonstrated that the classification model using LIBS combined with FA-PNN could realize better classification accuracy.

## 1. Introduction

Metal structural materials are commonly used in critical areas such as buildings, bridges, machinery, power stations, and aerospace [1,2,3,4,5], and the fields of application are increasingly broad and demanding with the emergence of industry and population growth, and must be designed and produced with high strength, high rigidity, excellent durability, and corrosion resistance to fulfill the growing demand [6,7]. While under prolonged utilization and loading, structural materials are exposed to irradiation, heat, mechanical stress, moisture, and other factors. These changes, which affect the internal microstructure and chemical composition of the material [8], cause performance degradation or even failure and may cause equipment downtime and major safety accidents. The aging grade is a crucial indicator for determining the level of structural material failure in the industrial safety evaluation system. Cutting out specified portions of the material and then employing metallographic microscopy for off-line examination [9] is the traditional method for evaluating the aging grade of structural materials. This method is excessively complex for sample pre-processing, takes a long time, and can destroy the material. Therefore, it is essential to classify structural materials and estimate their level of aging in order to improve equipment maintenance.

Traditional material aging grade research primarily focuses on the characterization of material properties, such as mechanical experiments to establish the relationship between force and the deformation of materials, in order to evaluate the mechanical properties of materials, including elasticity, plasticity, and hardness [10]. However, the method is unable to account for the influence of factors, including the internal microstructure, surface condition of materials and material properties, and the material is harmed and rendered useless. The structural and thermal properties of materials are evaluated using metallographic and thermal analysis in addition to mechanical experimental methods [11]. However, this approach necessitates sample preparation, which can result in sample damage and micro-temperature fluctuations that alter the material’s properties. Moreover, this renders it difficult to analyze and evaluate the materials. Spark direct reading spectrometry (SDRS) provides precise information and analytical tools for the study of material aging by identifying and quantifying changes in the elemental composition of materials [12]. However, the SDRS method can only be used for analyzing elements with higher content in metal materials, and samples must be polished repeatedly before testing so that their surface smoothness satisfies specific standards. This will result in some errors and uncertainties.

Laser-induced breakdown spectroscopy (LIBS) is an atomic spectroscopy analysis technology for elemental analysis of materials. Its principle is to focus a laser pulse on the sample’s surface to create a high-temperature plasma [13], collecting the plasma’s emitted spectrum and analyzing it. As a result of its advantages of rapid analysis, non-destructive measurement, lack of requirement for sample preparation, high sensitivity to low atomic weight elements, and long-distance measurement capabilities [14], it differs from conventional analytical technologies. Additionally, it can be used to detect elements in different states of matter, such as solids, liquids, gases, aerosols, etc. [15,16] Thus, LIBS has found widespread application in a variety of fields, such as environmental monitoring [17], biomedical applications [18], archaeological research [19], drug applications [20], extraterrestrial detection [21], hazardous material identification [22], nuclear industry [23], and geological material characteristics [24]. Furthermore, LIBS is also utilized to identify insulating faults in power supplies for medium-voltage applications [25].

The range of applications for machine learning (ML) has been growing quickly in recent years. It has been extensively researched and used in the field of material analysis combined with LIBS analysis technology, such as in the application of measuring material composition [13,26,27]. Moreover, the related physical and chemical properties (i.e., matrix effects) are different due to the various compositions of various materials. The laser ablation process and plasma characteristics can be impacted by these properties, according to earlier research [28,29]. Therefore, mechanical characteristics, elemental quantitative analysis, and microstructure analysis also can be performed using LIBS technology. Qiu et al. [30] used random forest regression to determine the content of elements in the sample. The results demonstrated that this approach reduced the detection limit, with a relative error of 0.02 wt.%. Shaik et al. [31] established a crude oil pipeline life prediction method based on the historical detection data of oil and gas fields and using the feedforward back propagation network (FFBPN). The research findings demonstrate that the crude oil pipeline life prediction model based on FFBPN has higher accuracy and better robustness than the published model, as measured by the maximum Coefficient of R^2^ and MSE. To study various aging grades of T91 steel samples, Lu et al. [32] combined laser-induced breakdown spectroscopy (LIBS) with support vector machines (SVM). The results showed that using multiple linear strengths and the average linear strength ratio as input variables considerably enhanced the model’s performance. However, this method does not take into account the crucial characteristics that differentiate between different age levels and simply assesses the aging level with a limited number of measurement points. Bakthavatchalam et al. [33] suggested an artificial neural network technique based on experimental datasets to forecast the relative thermophysical characteristics of the measured nanofluid. Temperature, concentration, size, and time were the model’s inputs, while the thermophysical properties were the model’s output. The results indicate that the R^2^ value is close to 1.0. Sanjana et al. [34] classified seven different types of contaminated silicone rubber insulators using machine learning technology with LIBS assistance. According to the findings, classification accuracy of LightGBM was 97.43%. Bellou et al. [35] used principal component analysis (PCA) and laser-induced shock spectroscopy (LIBS) to classify olive oil samples while researching the effects of experimental conditions on plasma properties. According to the findings, classification performance using appropriate algorithms is improved when experimental conditions are better. Gold ore formed as pressed particles from crushed bulk samples was classified using LIBS and principal component analysis (PCA) by Daniel Diaz et al. [36] The aforementioned research indicates that the combination of LIBS technology and machine learning has a lot of potential applications in the fields of material classification, element quantitative analysis, and mechanical performance research. However, no one has discovered a connection between artificial intelligence and the estimation of material aging degree. To accomplish a thorough characterization of material service behavior, it is essential to investigate the method of disclosing the multidimensional properties and degree of aging of structural materials based on machine learning.

In this study, a time-frequency feature extraction method using STFT and a deep feature mining method based on a similarity measurement were proposed to solve the challenge of traditional approaches’ limited capacity to predict aging time due to feature similarity retrieved from aging materials. A multitask model of a probabilistic neural network based on bionics algorithm optimization is developed, which can simultaneously realize material classification and aging time prediction, and the best optimization algorithm is selected after comparison. The framework is as follows: (1) establish an experimental system to collect spectral data of samples, LIBS spectral data is pre-processed to enhance the accuracy and stability of the data; (2) take features from pre-processed spectra using principal component analysis (PCA) and short-time Fourier transform (STFT), compare them to choose the best features, and explain the PCA’s limitations for extracting features from aging materials; (3) carry out probabilistic neural network (PNN) analysis and parameter optimization to categorize structural materials and forecast various aging levels based on the retrieved LIBS spectral feature data.

## 2. Experimental Setup and Samples

### 2.1. Experimental Setup

**Figure 1:**
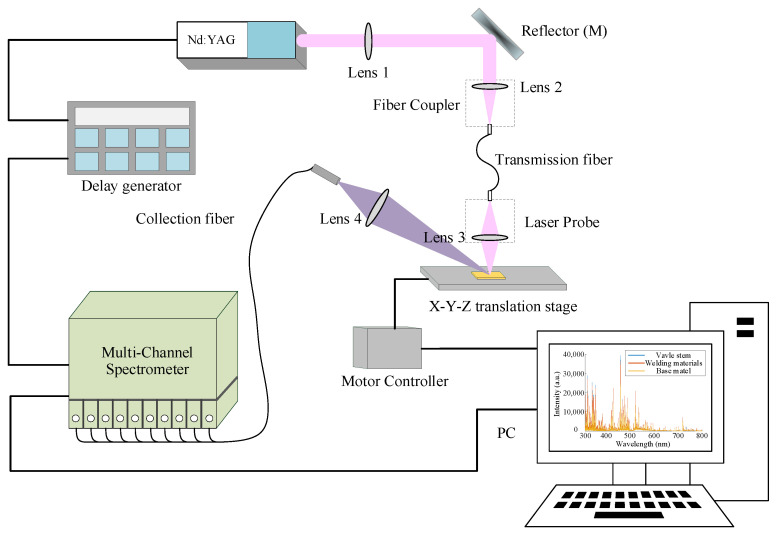
Schematic diagram of the experimental setup.

As shown in Figure 1, this experiment utilizes a fiber optic LIBS (FO-LIBS) system (this system was self-made, details can be viewed in our previous work [4]) with a single lens laser probe. A laser beam with a wavelength of 1064 nm and a pulse duration of 10 ns is produced by a Q-switch Nd:YAG pulse laser (GKNPS-1064-15-10, Beijing GK Laser Technology Co., Beijing, China) when it is operating at a repetition rate of 1 Hz. The main transmitted beam is reflected by the reflector M into the transmission fiber by the fiber coupler. A silica core and polyester cladding were found in the multi-mode transmission fiber used in the experiments (3 m length, 0.8 mm core diameter, 0.37 numerical aperture, and 1 GW/cm^2^ damage threshold for 10 ns pulse laser), which offers superior VIS/IR transmission for transmitting both laser beam and plasma emission signals.

Utilize a fiber coupler to guide the laser beam into the fiber. The laser beam incident on the fiber coupler operates at an energy of 40 mJ energy for each pulse to ensure the safe use of the transmission fiber, obtaining approximately 35 mJ of laser output from the fiber output end face. An ablation hole with a diameter of around 300 μm and an irradiance of 495.4 mJ/mm^2^ forms on the sample surface. The imaging principle is employed in the laser probe to focus the output laser. A 16 mm focal length aspherical lens (ACL25416U, Thorlabs Inc., Newton, NJ, USA) is used at the output end face of the transmission fiber to map the laser spot image of the fiber end face onto the sample surface in order to produce plasma. The sample can be moved and changed by manipulating the x, y, and z axis translation mechanisms on the platform, ensuring that each single pulse laser ablates a different position.

The plasma emission is guided by another convex lens (L4) with a 25 mm focal length into the collection fiber, which is connected to a spectrometer (Multi-channel spectrometer, spectral range 180–1064 nm, resolution: <0.12 nm, Avantes Inc., Apeldoorn, the Netherlands) to steadily obtain plasma emission. Optimize time delay to 1μs to avoid continuous emission and the integration time is set to 1 ms. In addition, a digital delay generator was applied to record the time series triggering laser and spectral acquisition.

### 2.2. Samples

The necessary information on the experimental samples, including name, size, and aging time, are given in Table 1 below. The valve stem is a component with the shape of a metal rod that is widely used in various industrial valves and pipeline systems to control and regulate the flow of fluid medium [37]. The welding material refers the metal filler substance utilized during the welding process to correct joint forms, fix faults in welded joints, and guarantee joint strength [38]. The base metal indicates the metal or alloy used in welding, casting, forging, processing, and other operations, which provides the majority of the transfer strength and bearing capacity in welded joints [39]. In this study, the material samples’ alloy information was as follows: valve stem, 15Cr12WNiMoV martensitic stainless steel, base metal, 316LN stainless steel (022Cr17Ni13Mo2N); and welding material, 308L stainless steel (06Cr20Ni11). The chemical composition of these materials is shown in Table 2. Long-term exposure to the conditions of high pressure, high temperature, and high torque causes changes in the microstructure and chemical characteristics of the aforementioned materials, which results in material aging and diminished mechanical properties. Therefore, it is required and crucial to establish a model based on machine learning to analyze the mechanism of material aging in order to improve the safety, stability, and service life of industrial equipment.

The obtained material samples have been processed in a high-temperature environment to model how the material samples would age under actual working environments. The well type air circulation uniform temperature furnace is used for the accelerated thermal aging test. Temperature for heat aging: 400 °C. Accelerated simulation experiments can reduce the experimental cycle, enhance data accuracy, and comprehensively evaluate the materials’ state of aging [40]. This will make it easier to evaluate the durability and stability of materials in actual-world circumstances, providing better theoretical guidance for engineering applications.

**Figure 2:**
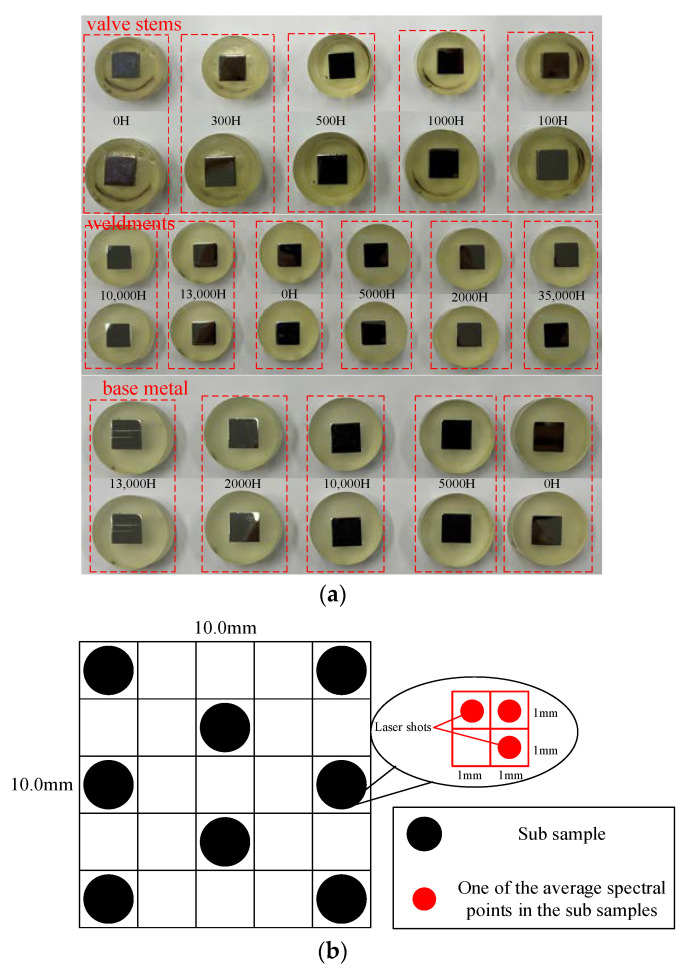
Samples and spectral data collection. (**a**) Experimental samples, (**b**) a method to increase the number of samples and spectral data set.

A photo of the samples is presented in Figure 2a. The material samples are separated into 5 × 5 grid sub sample areas in order to make better use of the samples. Each grid has a size of 2mm×2mm, representing a sub sample (Figure 2b). Three grids (red dot areas) were randomly selected from sub samples divided into the four grids, then a 3D mobile platform was used to perform 10 laser ablations at the center point of the selected sub sample grid and collect data. The average of 10 measurements was the spectrum of that point. Moreover, the average results of the three spectra were recorded as the spectral data of one sub sample. The quality and dependability of spectrum signals can be enhanced by reducing the impact of random noise through the use of multiple measurement results. Likewise, in order to prevent thermal and corrosion effects that might overlap the ablation range of nearby laser sites and affect the outcomes of the analysis, 8~9 grids were chosen from a total of 25 grids for ablation (8~9 sub samples). Finally, the sample spectra of various materials were gathered. Table 3 lists the number of sample spectra obtained and divides the dataset.

By employing this method, additional spectrum information on aging materials was gathered to give a substantial amount of trustworthy data for model development, and sample non-uniformity’s influence on measurement outcomes was minimized by ablation at various points of sub samples. The sample spectra of different materials were then obtained. Table 2 lists the number of sample spectra that were obtained and separates the dataset.

## 3. Data Analysis

**Figure 3:**
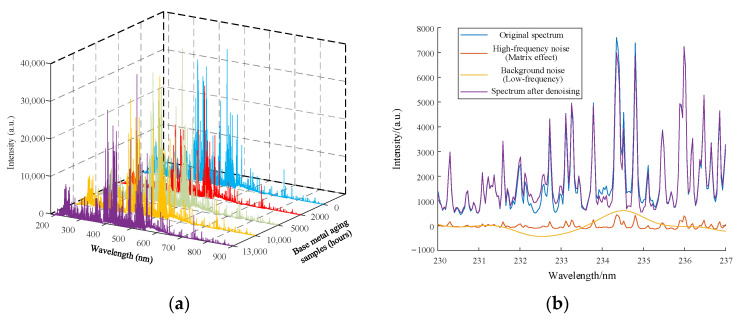
Samples spectrum. (**a**) Spectrums of aged samples, (**b**) waveform with original spectrum, high- and low-frequency noise, and denoised spectrum.

With each material containing sub samples at various aging levels, three material samples were examined using LIBS. The valve stem had age samples that had been operated for 0, 100, 300, 500, and 1000 h. The aging samples used for the welding material had service times of 0, 2000, 5000, 10,000, 13,000, and 35,000 h. Aging samples with aging times of 0, 2000, 5000, 10,000, and 13,000 h were included in the basis material. A total of 32 samples (sample library) were tested, and 250 spectral data were collected, each containing 20,480 pixels. As a result, the experimental dataset obtained was 250 × 20,480, using the spectrum of the base metal’s aging sample as an illustration, as depicted in Figure 3a. During measurement, a number of variables, including the matrix effect, self-absorption effect [41], gate delay, and environmental conditions, etc., had an impact on the LIBS spectra. Among them, high-frequency noise brought on by matrix effects, optical interference, and other reasons can result in abrupt peaks or dips in the spectrum, which appear as quick changes in light intensity. Spectral analysis’ precision and dependability may be impacted by this high-frequency noise. Low-frequency noise can generate baseline shifts in the spectrum that are continuous or smooth due to a self-absorption effect and spectrometer noise, which can also affect the analysis. As shown in Figure 3b, it can be clearly seen that high-frequency noise is caused by a matrix effect and low-frequency noise is caused by a self-absorption effect. Therefore, it is necessary to preprocess the LIBS spectral data in order to increase the analysis’s accuracy [42], as described in Section 3.1. Direct application of machine learning models might cause problems with convergence due to the large dimensionality of spectral input, making it difficult to improve the model’s accuracy. In Section 3.2, the pre-processed spectral data were used to extract features and different feature extraction algorithms were compared. The extracted feature dataset was divided, and probabilistic neural network algorithms were used to classify various types of materials and varying degrees of aging under the same material.

### 3.1. Spectral Data Pre-Processing

**Figure 4:**
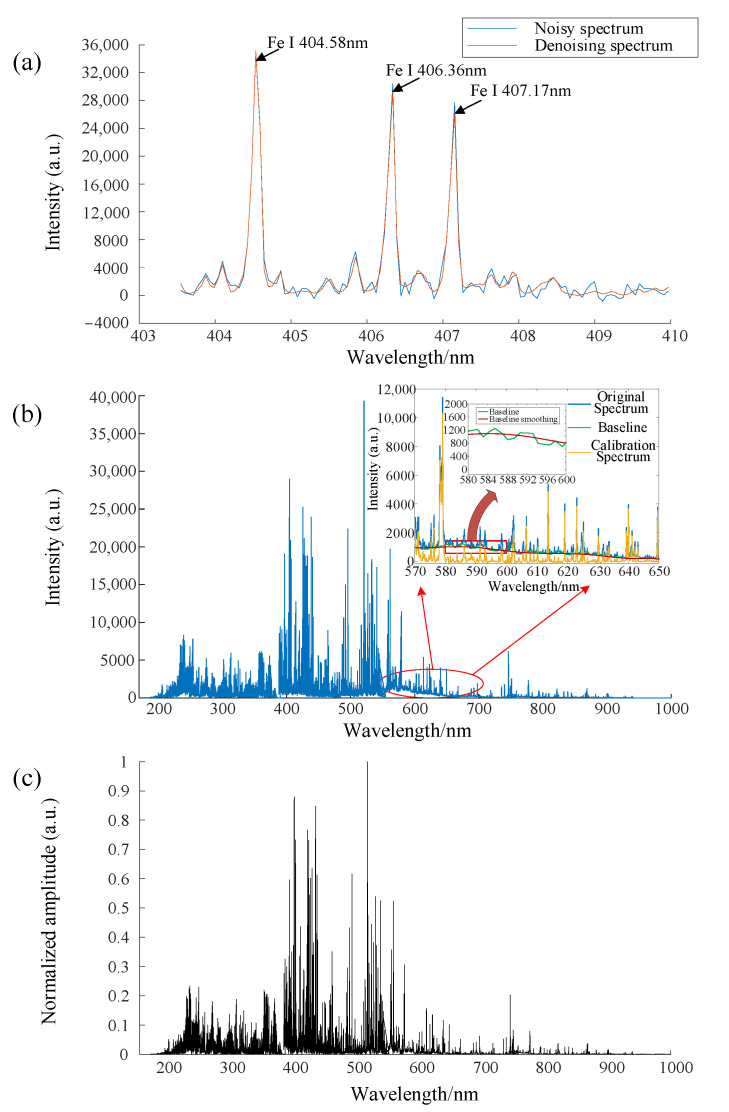
Pre-processing of spectral data. (**a**) Wavelet threshold noise reduction, (**b**) baseline calibration, (**c**) normalization.

There will typically be some spectrum changes between the observations of each pulse due to the non-uniformity of the sample surface, interference from the environment, and variations in laser energy. These data fluctuations can be decreased with appropriate data pretreatment. In this work, the pre-processing of the spectral data includes wavelet threshold noise reduction (Figure 4a), baseline calibration based on the segmented feature extraction method (Figure 4b), and maximum-minimum normalization processing (Figure 4c).

In the process of using wavelet threshold denoising, the spectral was ultimately decomposed into four layers using db6 wavelet bases and fixed thresholds after testing the denoising effects of various wavelet bases and decomposition sizes. A soft threshold function, which is an improvement over a hard threshold function and has better smoothness in denoising, is chosen among them by the threshold processing. The soft threshold function is described as follows in this article:

(1) $$\overset{-}{w_{j,k}}=\left\{\begin{array}{c} sgn\left(w_{j,k}\right)\left(w_{j,k}-\lambda \right)\;,\left|w_{j,k}\right|>\lambda \\ 0,\;\left|w_{j,k}\right|<\lambda \end{array}\right.$$

where sgn() is a sign function, $w_{j,k}$ is the wavelet coefficient before threshold processing, and $\bar{w_{j,k}}$ is the wavelet coefficient after threshold processing, *λ* represents the threshold. The commonly used threshold is:

(2) $$\lambda =\sqrt{2lnX}$$

In the formula, *X* is the number of wavelength points in the spectrum. The threshold selected for this study, *λ*, is 3.15.

The segmented feature value extraction method was used in this work as the baseline correction technique for spectral data.

Step 1: Equally divide the LIBS spectrum into *N* sets of data point groups.

(3) $$N=\frac{Wavelength}{number}=\frac{20,480}{2048}=10$$

Step 2: Calculate the minimum spectral intensity of each data point group as the eigenvalues of the spectrum in that data group.

(4) $$min{\left\{A\left(\alpha_{i}\right)\right\}}_{j},j=1,2,3\cdots N$$

Step 3: Subtract the corresponding eigenvalues of each data point group, and finally concatenate all data point groups to obtain the baseline corrected spectrum.

The maximum-minimum approach, which sets the spectrum’s intensity values to [0, 1], was utilized in this study to normalize the spectral data. According to the following normalizing formula:

(5) $$y=\frac{y-y_{min}}{y_{max}-y_{min}}$$

In the formula, *y* stands for the intensity values of the group of spectra at different wavelengths, while $y_{min}$ and $y_{max}$ stand for the intensity values of the group’s spectral data’s minimum and maximum values.

### 3.2. LIBS Spectral Feature Extraction and Similarity Metric

Directly applying machine learning models can cause difficulties with convergence and other issues due to the high dimensionality of the spectrum input. Feature extraction on spectral data should be carried out in order to enhance the model’s performance and interpretability [43]. Principal component analysis (PCA), a statistical technique for dimensionality reduction for high-dimensional datasets, has been applied frequently in the analysis of LIBS spectral data [44]. Its basic idea is to reduce high-dimensional data to a set of principal components (PCs) by projecting it downward into a low-dimensional subspace. The variance contained in each PC is used as the eigenvalues of the spectral dataset, which serve as inputs to the neural network. However, PCA is not without flaws, including the inability to handle nonlinear data, the disregard for non-variance information (such as correlation and outliers), the high processing cost, and perhaps the lack of interpretability of extracted features. Data time-frequency processing and analysis methods have drawn increasing amounts of attention in recent years and have developed into effective tools for time-varying non-stationary signals. A well-known technique for time-frequency analysis, the short-time Fourier transform (STFT), is frequently employed for feature extraction [45]. The STFT overcomes the limitations of the Fourier transform, which include its poor performance on abrupt and non-stationary signals as well as its inability to characterize the local properties of signals in the time domain. STFT can be used to visualize data in the time spectrum (or time scale) domain and intuitively observe the time-frequency characteristics of the data, while the principal components extracted by PCA may not have intuitive interpretability. In this study, the LIBS spectrum data features were extracted using STFT, and similarity tests were performed on the extracted spectral feature [46,47] (Formulas (1) and (2)). In various material service behavior situations, the results revealed a single change, and the similarity measurement results of the material spectral feature were used as input for the multi-classification deep learning model. This study applies PCA to extract feature values (feature 1) and the multi-frequency spectral feature extraction based on STFT (feature 2). These feature values are then inputted into the same network for classification prediction and comparison.

(6) $$X^{*}=(X-m)/s$$

(7) $$d=\sqrt{\sum_{k=1}^{n}{\left(\frac{x_{1k}-x_{2k}}{s_{k}}\right)}^{2}}$$

where X* is the normalized value, X is the value before normalization, m is the mean of the components, s is the standard deviation of the components,$x_{1k}$, $x_{2k}$ are the standard sample data and the measured sample data, and d is the normalized Euclidean distance. The similarity measure based on Euclidean distance measures the distance between two vectors by calculating the square root of the sum of the squares of the differences between their respective dimensions. After measuring the similarity of the characteristics of aging materials, the degree of aging or similarity between different materials can be more accurately evaluated, which can help identify common patterns or related features in aging materials. This is crucial for predicting material properties, evaluating reliability, and identifying potential aging and defect mechanisms.

### 3.3. PNN in LIBS

**Figure 5:**
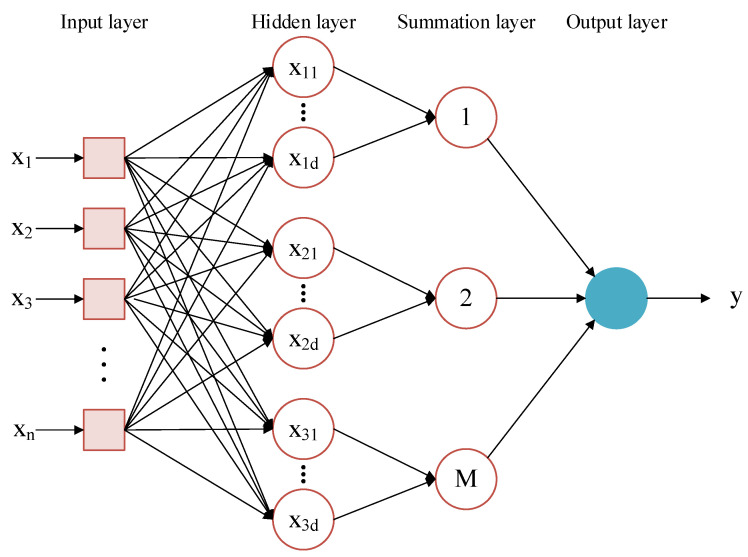
Network structure of PNN.

PNN is a form of feedforward network that combines density function estimation and Bayesian decision theory to classify samples based on radial basis function (RBF) networks [48]. Its network structure is shown in Figure 5. The input layer, hidden layer, summation layer, and output layer are the four components that make up the PNN network. The input layer is used to transfer information to the hidden layer and receive values from training samples, and the number of neurons is equal to the number of input variables. The hidden layer is a radial base layer with each neuron corresponding to a center, and the distance between the input vector and the center is determined. A scalar value is eventually returned. The performance of PNN will be impacted by the number of hidden layer neurons n, which should be configured in accordance with the particular application. The summation layer has M nodes, each of which represents a class. The summing layer has M nodes, each of which corresponds to a class. The decision-making process is determined using the summation layer’s competitive transfer function. Resulting from that, the output layer outputs the decision result, with only one 1 and all other results being 0. The output result of the classification that has the highest probability value is 1.

The activation function of each neuron in the hidden layer is given by the probability density function based on the Gaussian kernel, and the formula below describes the link between input and output determined by the jth neuron of class i:

(8) $$\phi_{ij}(x)=\frac{1}{{\left(2\pi \right)}^{\frac{1}{2}}\delta^{d}}e^{-\frac{(x-x_{ij}){\left(x-x_{ij}\right)}^{T}}{2\delta^{2}}}$$

where i = 1, 2, ⋯, M, M is the total number of classes in the training samples, $x_{ij}$ is the kth training sample belonging to the ith class of samples, d is the dimensionality of the sample vector, and *σ* is the smoothing parameter.

The summation layer takes the weighted average of the outputs of the hidden neurons belonging to the same class in the hidden layer:

(9) $$p(x|v_{i})=\frac{\sum_{j=1}^{N_{i}}\phi_{ij}}{N_{i}}=\frac{1}{N}\sum_{j=1}^{N_{i}}\frac{1}{(2\pi )\delta^{d}}e^{-\frac{(x-x_{ij}){\left(x-x_{ij}\right)}^{T}}{2\delta^{2}}}$$

where $v_{i}$ denotes the output of the ith category, $N_{i}$ is the total number of training samples of the $v_{ith}$ category, and the number of neurons in the summation layer is the same as the number of categories *M*.

Remove common elements and define the discriminant function as follows based on the input/output relationship between the hidden layer and the summing layer:

(10) $$g_{i}(x)=\frac{p(v_{i})}{N_{i}}\sum_{j=1}^{N_{i}}e^{-\frac{||x-x_{ij}|{|}^{2}}{2\delta^{2}}}$$

The greatest g(x) in the summation layer is selected as the output category in the output layer:

(11) $$y=Class\;of\;Max\left(g_{1},g_{2},\cdots ,g_{i}\right)=\mathrm{argmax}(g_{i}(x))$$

PNN has excellent adaptive learning and fault tolerance capabilities. The choice of parameters [49], such as smoothing parameter *σ*, the number of hidden layer nodes *n*, the hidden center vector *c* (the center vector of each pattern category), etc., affects how well the network structure performs. The value of *σ* is too small, which only serves as isolation for separately trained samples, and the value of *σ* is too large to fully distinguish details, and for different categories with unclear boundaries, the ideal classification effect may not be achieved, which is close to linear classification. In order to improve the accuracy of the network and achieve the best classification results, this research chooses bionics optimization algorithms (such as genetic algorithm (GA), particle swarm optimization (PSO), dragonfly algorithm (FA), etc.) to determine the smoothing parameter *σ* and the number of hidden nodes *n*. A classification model based on FA-PNN was ultimately chosen by contrasting various iterative optimization algorithms.

In this study, for multiple material classification and aging time estimation, LIBS spectral data and PNN algorithm are combined. (1) Use spectral feature sets to categorize the three materials. (2) Following the determination of the classification outcomes from (1), extract the material’s aging time feature dataset and arbitrarily split it into a 70% training set and a 30% testing set. To categorize material samples with various degrees of aging, the same PNN model as in step one is applied. (3) After utilizing optimization algorithms to optimize the structural parameters in PNN, create an FA-PNN model and perform the classification of the material aging time. Employ the data sets of distinct aging degrees of the other two materials as prediction sets to verify the generalization ability of the constructed model.

## 4. Results and Discussion

### 4.1. Spectra of Material Samples

**Figure 6:**
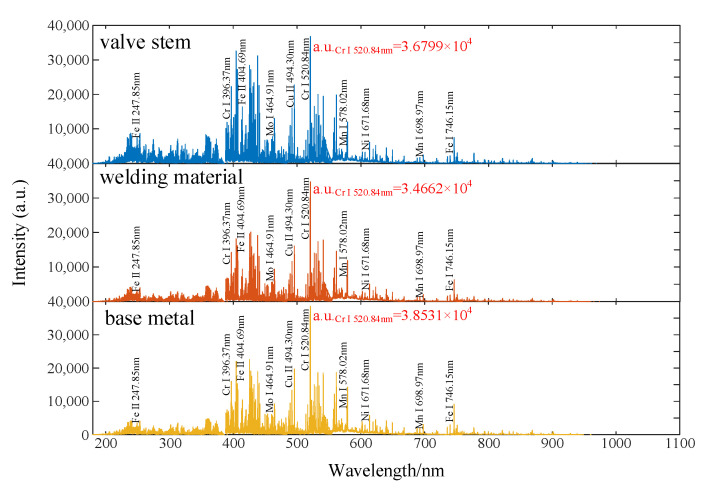
Spectra of the three material samples.

The LIBS spectra of the three material samples are shown in Figure 6. The spectra of all three material samples show the presence of the emission lines of Cr (396.38 nm, 520.84 nm, etc.), Fe (404.58 nm, 430.79 nm, etc.), Mo (386.41 nm, 466.29 nm, etc.), Ni (671.68 nm), and Mn (403.31 nm, 578.02 nm). The strength of these spectral peaks, however, varies greatly between various materials. For instance, Cr (520.84 nm) and Fe (430.79 nm) in the base metal are higher than the other two materials. In the spectrum of welding materials, the Cr/Fe ratio is approximately 2.798, whereas the Cr/Fe value in the base metal is roughly 1.658. It is important to note that these spectral data’s intensity and ratios play a crucial role in quantitative analysis and can also act as a defining characteristic for materials. However, due to the material’s constant element content and chemical makeup, which do not alter with the degree of aging, it is challenging to distinguish the same type of material under various levels of aging using the ratio of spectral intensity. In order to effectively capture the subtle changes in features between materials at various levels of aging, it is required to apply PCA and STFT feature extraction algorithms to extract the spectral data of aged materials. The features retrieved by PCA and STFT for material type categorization and aging time prediction will be assessed in the sections that follow.

### 4.2. Feature Data Selection

**Figure 7:**
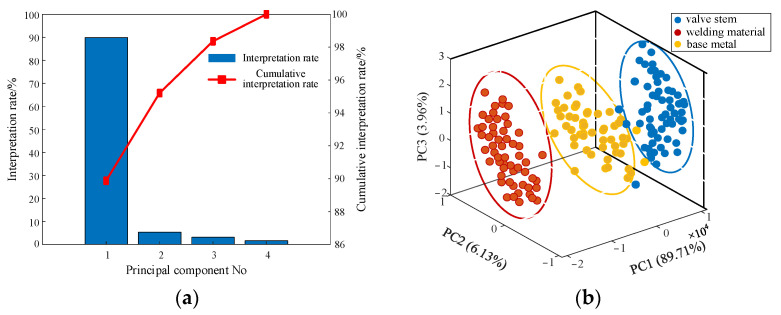
Principal component analysis for feature extraction. (**a**) Principal component contribution map, (**b**) principal component analysis score graph.

In this study, we conducted a PCA analysis using the entire spectrum of all training set spectral data, using the covariance matrix as its foundation. The PCA-extracted features (feature 1) and the STFT multi-band spectral feature extraction based on a similarity metric (feature 2) are input to the same network for ACC comparison to select the optimal features. Every principal component (PC) in PCA corresponds to a feature vector. The first of these feature vectors (PC1), which represents the direction with the highest variance, is followed by the PC2, representing the direction with the second-highest variance, and so on. When dimensioning the data, only the first k feature vectors with the highest variance are retained. A training set of 168 spectra was utilized for training the PCA model. Figure 7 depicts the training samples’ principal component score map and principal component contribution map. The first four PCs’ cumulative contribution rate, which may be used to represent the key information of the material spectrum, reached 99.8%, as can be shown in Figure 7a. As a result, the variance characteristic variables of the first four PCs were chosen to be the PNN model’s inputs, and this feature is noted as feature one. A point in Figure 7b represents a material sample, which describes the conversion of input spectral data into PC space. It is evident that the data points for the various types of material samples are clustered into distinct clusters, showing that the PCA approach can be used to extract and classify features from spectral data. Therefore, it is used to extract features from spectral data of materials with different degrees of aging.

**Figure 8:**
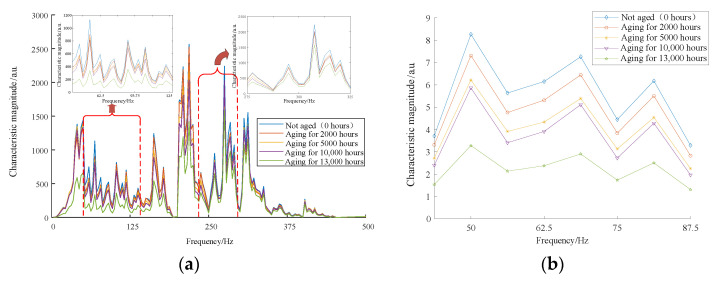
STFT for feature extraction. (**a**) STFT-based multi-band spectral feature extraction, (**b**) band map after similarity metric.

With the usage of STFT, the signal can be divided into a number of frequency bands, each of which holds the signal’s current frequency information. Feature extraction techniques can quickly get feature quantities that identify different material kinds from LIBS spectra of various materials. The features derived using PCA are insufficient to discriminate the degree of age because the LIBS spectra of the same material with various levels of aging are remarkably similar. As a result, from a frequency domain perspective, STFT can be used to analyze the spectrum, understand the intensity changes of spectrum signals at various frequencies, identify some frequency domain features in the signal, and then use these frequency domain features for feature quantity classification of aging degree. Figure 8a displays the extraction of multi-band spectral features based on STFT. The extracted spectral features in the feature frequency bands (31.25–125 Hz, 275–325 Hz) exhibit singularity changes under different material service behavior conditions after similarity measurement (Figure 8b), which is called feature two. 

Next, establish a PNN model using features one and two. In this study, the PNN models’ performance is measured using the accuracy of classification (*ACC*), which is the percentage of correctly classified samples across all samples in the model. The following is the calculating formula:

(12) $$ACC=\frac{\mathrm{Number}\;\mathrm{of}\;\mathrm{correctly}\;\mathrm{classified}\;\mathrm{samples}}{\mathrm{Total}\;\mathrm{number}\;\mathrm{of}\;\mathrm{samples}}\times 100\%$$

**Figure 9:**
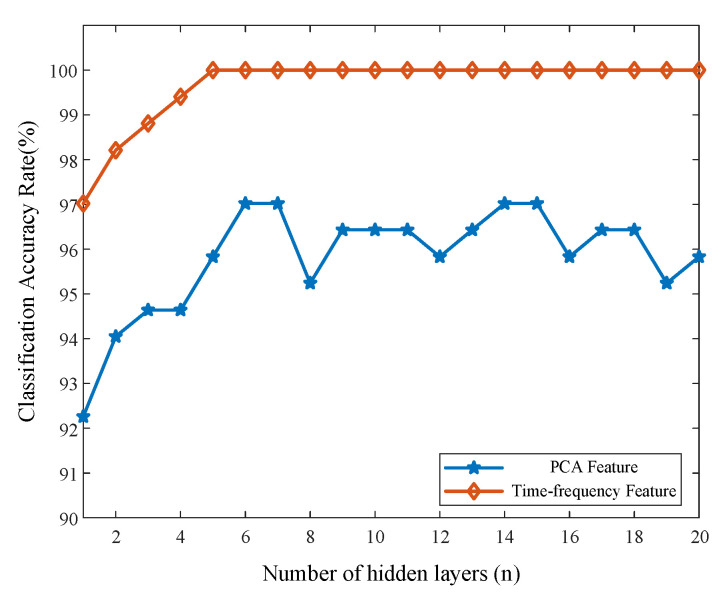
Effect of the number of hidden layers *n* on ACC in PNN model.

Figure 9 shows the *ACC* of the resulting model. When n changes from one to 20 and features one (PCA feature) and two (time-frequency feature) are utilized as inputs, the *ACC* of both reaches its maximum value when n is set to six. The *ACC*, based on the PNN model, fluctuates between 92.26% and 96.43%, with an average value of 95.8% when feature one is employed as the input variable. In the case of using time-frequency features as input variables, the ACCs are all 100% when *n* > 5. Feature two is higher than feature one from the start (*n* = 1), and the *ACC* of time-frequency features is higher than the *ACC* of *PCA* features for the average *ACC* corresponding to the overall *n*. The outcomes suggest that utilizing time-frequency features as input variables may be more effective than using PCA features as input variables in a probabilistic neural network classification model.

**Figure 10:**
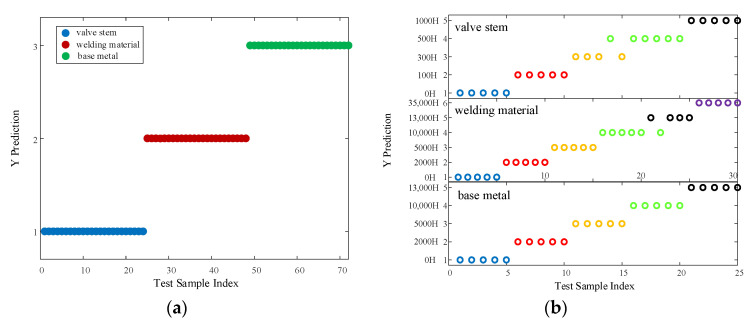
Material classification and aging time prediction based on feature two. (**a**) Material classification based on test sets, (**b**) prediction of different degrees of aging under the same material.

As a consequence, feature two was selected for training and prediction of the PNN model in the subsequent research. The test set was used to classify three materials with different degrees of aging, as shown in Figure 10. The *ACC* of the test set utilized for material classification was 100%, and the *ACC* of the test set with different degrees of aging under the same material was 96% for the valve stem; 96.67% for the welding material; and 100% for the base metal.

### 4.3. Optimization of PNN Structure

**Figure 11:**
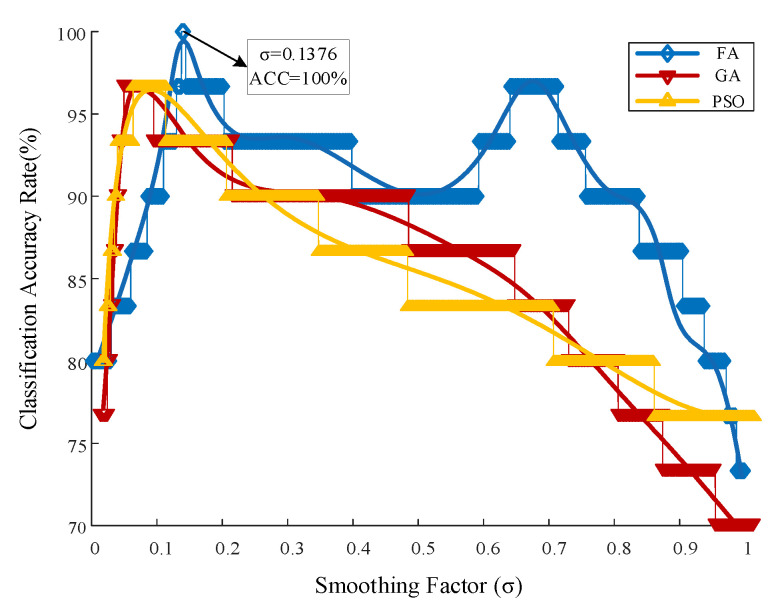
Effect of smoothing parameter *σ* on the classification accuracy of different aging degrees.

We considered establishing a PNN model based on the previously identified spectral data to classify materials and predict various aging durations. However, smoothing factor σ has a significant impact on the diagnostic results of PNN. The accuracy of PNN recognition is decreased by the inability to completely capture probabilistic characteristics because smoothing parameters are generally assumed $\sigma_{1}=\sigma_{2}=\cdots =\sigma$ in traditional PNN. After that, we applied the bionics algorithm to the probabilistic neural network’s parameters. Figure 11 displays the outcomes of the optimization using the GA, PSO, and FA optimization algorithms. It can be seen that the optimal parameter σ = 0.1376 is obtained when the PNN is optimized using FA, at which time the classification accuracy of different aging degrees reaches 100%. However, this involves utilizing GA and PSO to optimize PNN and beginning from iteration. ACC reaches the corresponding highest classification accuracy rate (96.67%) as σ increases. When compared to FA_PNN, ACC of GA_PNN and PSO_PNN is poor and, as a result, σ =0.1376 of FA _ PNN model was subsequently adopted for training and prediction.

**Figure 12:**
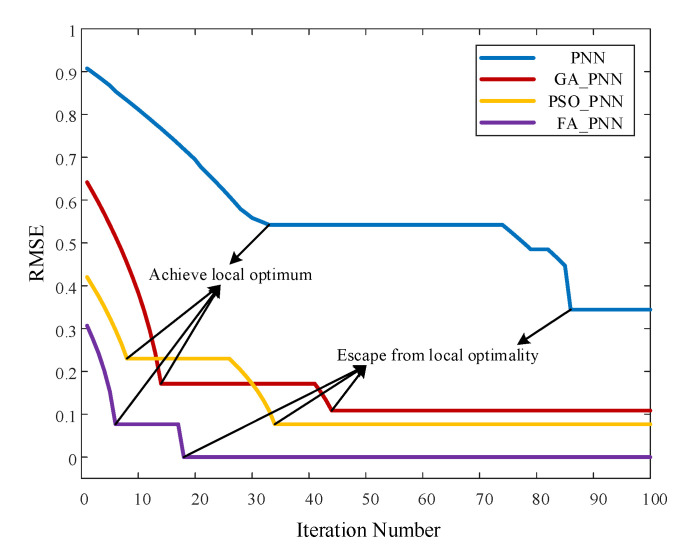
Iterative process of each optimization algorithm.

For each state, 55 sets of feature vectors were simultaneously extracted at random and trained individually on the PNN, GA_PNN, PSO_PNN and FA_PNN network. The optimization process of smoothing parameter *σ* is shown in Figure 12. The ordinate shows the root-mean-square deviation (RMSE) between the output value of the training sample and the actual value (label value), while the abscissa shows the number of iterations.

The graphic indicates that each optimization algorithm corresponds to the optimal *σ* and has its own local and global optima. See Table 4 for details.

The remaining 25 sets of feature vector groups for each aging state were then used as the test set and substituted into the trained network, respectively. The comparison of the experimental results is shown in Table 5.

**Figure 13:**
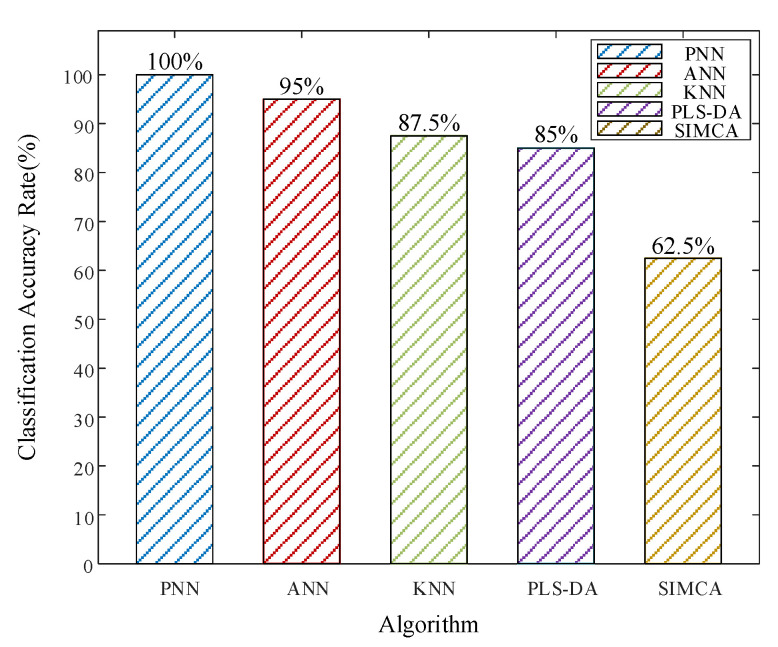
ACC of PNN, ANN, KNN, PLS-DA, SIMCA.

In addition, for classification evaluation, additional widely used stoichiometric techniques (PLSDA, SIMCA) and machine learning models (KNN, ANN) were applied. Figure 13 depicts the tested models’ optimal ACCs for each model. PNN, ANN, KNN, PLS-DA, and SIMCA all have ideal ACC values of 100%, 95%, 87.5%, 85%, and 62.5%, respectively. The results show that the PNN model has the best recognition ability among all the classification models tested.

### 4.4. Research on the Physical Mechanism of Aging

In power plants and other factories, structural materials are vulnerable to exposure to high temperatures, high humidity, stress, and radiation. Through phenomena such chain breakage, cross-linking, and oxidation, materials may have their surface characteristics, chemical composition, and physical structure altered [50]. A single surface of the valve stem sample was polished with 2000 grit sandpaper and a glass plate prior to the experiment, and hardness testing was carried out using a hardness tester (HXD-1000TMC/LCD), in order to investigate the relationship between the hardness and the spectral features of aging samples. The average hardness value for each sample was determined after three tests, as indicated in Table 6.

**Figure 14:**
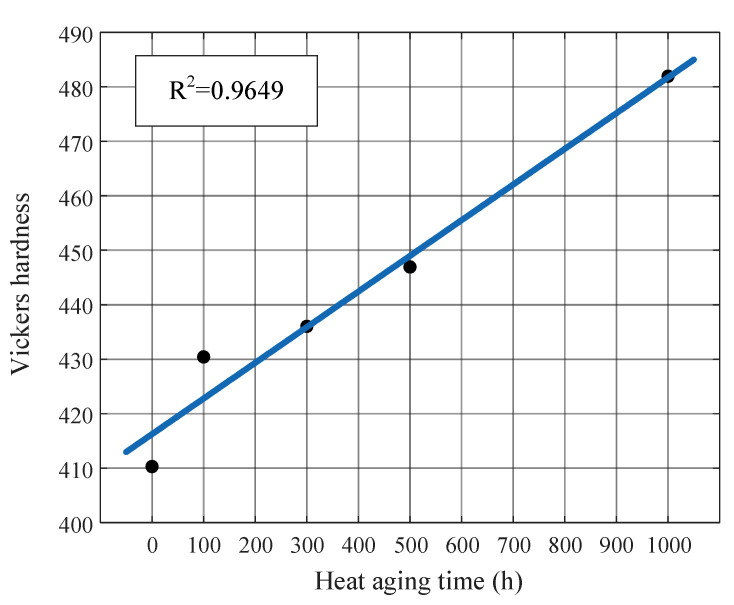
Relationship between aging time and surface Vickers hardness of valve stem aging samples.

It was discovered that there is a substantial linear link between the hardness of the valve stem material and its aging time, based on the relationship between the aging time of the valve stem aging sample and the surface’s Vickers hardness (Figure 14). It is possible to think that as material ages, its surface hardens, which may reflect the issue of the valve stem becoming brittle with increased use and splitting easily. The ability to withstand external damage increases with the material’s surface hardness. Since less plasma is formed during the ablation process of a laser pulse under the same laser irradiance, there is more concentrated energy and a lower energy absorption efficiency, which causes the plasma temperature to rise during the same time window [51]. Thus, certain properties (such as relative intensity, emission wavelength intensity, peak height/position/area, spectral line width, spectral power/energy, etc.) of spectrum signals alter with increasing levels of aging, and the alterations in properties under various levels of aging for the same material are relatively subtle and are not visible to the naked eye or by using basic evaluation indications. A workable method is to extract features, enhance feature discrimination, and create a material aging analysis model utilizing machine learning and data mining.

## 5. Summary and Prospective

In this paper, a new method based on a probabilistic neural network model combined with LIBS spectral data for multi-classification of material samples and aging degree is proposed. The raw spectral data are firstly pre-processed (noise reduction, baseline calibration, and normalization), and the spectral data after PCA dimensionality reduction and multi-band data under time-frequency analysis are selected as spectral features. The ACCs of the neural network model based on different numbers of hidden neurons and iteration algorithms are compared. Experimental results show that the number of hidden neurons that best fits the neural network model is six, the optimal iteration algorithm is FA, and the value of the optimal parameter *σ* under FA_PNN is determined. We also compared the ACC of the models built using PCA features and time-frequency features as input variables of the test set, and the results show that the recognition ability of the model built with time-frequency features as input variables is better than that of the model built with PCA features as input variables. The optimal ACCs of neural networks and other well-known models in spectral analysis (ANN, PLS-DA, KNN, and SIMCA) on the test set were also evaluated, and the results showed that only the probabilistic neural network model achieved 100% ACC, which indicates the success of LIBS combined with probabilistic neural networks in the identification of material types and different aging levels. Through the analysis of historical data and real-time monitoring data in real-time practice, this study effort provides a prediction and early warning of material aging time, offering a direction for material aging monitoring and evaluation. It has significant practical significance for extending equipment life and doing preventative maintenance. Meanwhile, the efficacy of simulation experiments for thermal aging and corrosion can be improved while material aging time prediction based on time-frequency cfharacteristics and the FA-PNN model could provide guidance for simulation experiments, such as optimizing experimental parameters, determining experimental time, and choosing suitable aging conditions.

In our future research, LIBS will be used for quantitative analysis of material samples, and we will explore the use of emerging technologies like attention mechanisms or graph convolutional networks in PNN, as well as the application of multi-scale Gaussian kernels to solve the issue of significant spatial scale variations.

## Figures and Tables

**Table 1:** Sample information.

Material Name	Number of Samples	Sample Size	Aging Degree (Hours)
Valve Stem	10	10 × 10 × 4 mm	0, 100, 300, 500, 1000
Welding Material	12	10 × 10 × 4 mm	0, 2000, 5000, 10,000, 13,000, 35,000
Base Metal	10	10 × 10 × 4 mm	0, 2000, 5000, 10,000, 13,000

**Table 2:** Chemical composition of materials.

Material Name	Mass Fraction of Elements, wt.%										
	C	Si	Mn	P	S	Ni	N	Cr	Cu	W	Fe
Valve Stem	0.15	0.8	0.9	<0.03	<0.025	0.5	—	11.2	0.5	0.7	~83
Welding Material	0.016	0.32	1.33	0.015	0.011	10.30	—	19.88	0.065	—	Bal.
Base Metal	0.011	0.24	1.30	—	—	13.12	0.122	17.18	—	—	Bal.

**Table 3:** Spectral data set of material samples.

**Material Name**	**Total Number of Spectra**	Materials Classification	**Training Set**	**Test Set**	Aging Prediction	**Training Set**	**Test Set**
Valve Stem	80		56 (7 samples)	24 (3 samples)		55 (5 aging levels)	25 (5 aging levels)
Welding Material	90		56 (8 samples)	28 (4 samples)		60 (6 aging levels)	30 (6 aging levels)
Base Metal	80		56 (8 samples)	24 (3 samples)		55 (5 aging levels)	25 (5 aging levels)

**Table 4:** Optimal parameters and performance evaluation.

	PNN	GA_PNN	PSO_PNN	GA_PNN
Optimal *σ*	0.5	0.0499	0.0734	0.1376
Number of Iterations at Local Optimal Time	33	14	8	6
Local Optimal RMSE	0.5423	0.1715	0.2301	0.0767
Number of Iterations at Global Optimal Time	86	45	34	17
Global Optimal RMSE	0.3443	0.1085	0.0767	0

**Table 5:** Comparison of experimental results.

Algorithm	Training Samples			Test Samples			Running Time/s
	Total Sample	Correct Sample Identification	Correct Rate/%	Total Sample	Correct Sample Identification	Correct Rate/%	
PNN (σ=0.5)	170	155	91.1764	80	74	92.50	1.8750
GA_PNN (σ=0.0499)	170	170	100	80	77	96.25	306.7247
PSO_PNN (σ=0.0734)	170	170	100	80	77	96.25	183.1538
FA_PNN (σ=0.1376)	170	170	100	80	80	100	7.3597

**Table 6:** Hardness test of aging samples.

Number	Operation Hours	Hardness Test Value (Vickers Hardness)			Average
		First Test	Second Test	Third Test	
0	0.5	411.8	407.3	411.8	410.3
1	33	423.2	430.4	437.7	430.4333
2	0.5423	435.2	432.8	440.1	436.0333
3	86	458.1	442.6	440.1	446.9333
4	0.3443	474.3	477.1	494.4	481.9333

## Data Availability

The data that support the findings of this study are available from the corresponding author upon reasonable request.

## References

[1] Gonzalez-Gutierrez J., Cano S., Schuschnigg S., Kukla C., Sapkota J., Holzer C. (2018). Additive Manufacturing of Metallic and Ceramic Components by the Material Extrusion of Highly-Filled Polymers: A Review and Future Perspectives. Materials.

[2] Lesiuk G., Smolnicki M., Rozumek D., Krechkovska H., Student O., Correia J., Mech R., De Jesus A. (2020). Study of the Fatigue Crack Growth in Long-Term Operated Mild Steel under Mixed-Mode (I + II, I + III) Loading Conditions. Materials.

[3] Zykova A.P., Tarasov S.Y., Chumaevskiy A.V., Kolubaev E.A. (2020). A Review of Friction Stir Processing of Structural Metallic Materials: Process, Properties, and Methods. Metals.

[4] Qiu Y., Wu J., Li X., Liu T., Xue F., Yang Z., Zhang Z., Yu H. (2018). Parametric Study of Fiber-Optic Laser-Induced Breakdown Spectroscopy for Elemental Analysis of Z3CN20-09M Steel from Nuclear Power Plants. Spectrochim. Acta Part B At. Spectrosc..

[5] Williams J.C., Boyer R.R. (2020). Opportunities and Issues in the Application of Titanium Alloys for Aerospace Components. Metals.

[6] Hsissou R., Seghiri R., Benzekri Z., Hilali M., Rafik M., Elharfi A. (2021). Polymer composite materials: A comprehensive review. Compos. Struct..

[7] Saleh B., Jiang J., Fathi R., Al-hababi T., Xu Q., Wang L., Song D., Ma A. (2020). 30 Years of Functionally Graded Materials: An Overview of Manufacturing Methods, Applications and Future Challenges. Compos. Part B Eng..

[8] Fournier B., Maxime S., Barcelo F., Rauch E., Renault-Laborne A., Cozzika T., Dupuy L., Pineau A. (2009). Creep-Fatigue Interactions in a 9 Pct Cr1 Pct Mo Martensitic Steel: Part II. Microstructural Evolutions. Metall. Mater. Trans. A-Phys. Metall. Mater. Sci..

[9] China N. D. A. R. (2004). Power Plant Metallography Inspection and Assessment Guideline.

[10] Guo Y.B., Liu C.R. (2002). Mechanical Properties of Hardened AISI 52100 Steel in Hard Machining Processes. J. Manuf. Sci. Eng..

[11] Król M., Tański T., Snopiński P., Tomiczek B. (2017). Structure and Properties of Aluminium–Magnesium Casting Alloys after Heat Treatment. J. Therm. Anal. Calorim..

[12] Cremers D.A., Radziemski L.J. (2017). Laser Plasmas for Chemical Analysis. Laser Spectroscopy and Its Applications.

[13] Noll R., Fricke-Begemann C., Brunk M., Connemann S., Meinhardt C., Scharun M., Sturm V., Makowe J., Gehlen C. (2014). Laser-Induced Breakdown Spectroscopy Expands into Industrial Applications. Spectrochim. Acta Part B At. Spectrosc..

[14] Singh J., Kumar R., Awasthi S., Singh V., Rai A.K. (2017). Laser Induced Breakdown Spectroscopy: A Rapid Tool for the Identification and Quantification of Minerals in Cucurbit Seeds. Food Chem..

[15] Zhang D.C., Hu Z.Q., Su Y.B., Hai B., Zhu X.L., Zhu J.F., Ma X. (2018). Simple Method for Liquid Analysis by Laser-Induced Breakdown Spectroscopy (LIBS). Opt. Express.

[16] Vanselow C., Stöbener D., Kiefer J., Fischer A. (2019). Revealing the Impact of Laser-Induced Breakdown on a Gas Flow. Meas. Sci. Technol..

[17] Effenberger A.J., Scott J.R. (2010). Effect of Atmospheric Conditions on LIBS Spectra. Sensors.

[18] Šindelářová A., Pořízka P., Modlitbová P., Vrlíková L., Kiss K., Kaška M., Prochazka D., Vrábel J., Buchtová M., Kaiser J. (2021). Methodology for the Implementation of Internal Standard to Laser-Induced Breakdown Spectroscopy Analysis of Soft Tissues. Sensors.

[19] Ruan F., Zhang T., Li H. (2019). Laser-Induced Breakdown Spectroscopy in Archeological Science: A Review of Its Application and Future Perspectives. Appl. Spectrosc. Rev..

[20] Tiwari P.K., Rai N.K., Kumar R., Parigger C.G., Rai A.K. (2019). Atomic and Molecular Laser-Induced Breakdown Spectroscopy of Selected Pharmaceuticals. Atoms.

[21] Qiao S., Ding Y., Tian D., Yao L., Yang G. (2015). A Review of Laser-Induced Breakdown Spectroscopy for Analysis of Geological Materials. Appl. Spectrosc. Rev..

[22] Gottfried J.L., De Lucia Jr F.C., Munson C.A., Miziolek A.W. (2007). Double-Pulse Standoff Laser-Induced Breakdown Spectroscopy for Versatile Hazardous Materials Detection. Spectrochim. Acta Part B At. Spectrosc..

[23] Wu J., Qiu Y., Li X., Yu H., Zhang Z., Qiu A. (2020). Progress of Laser-Induced Breakdown Spectroscopy in Nuclear Industry Applications. J. Phys. D Appl. Phys..

[24] Shi M., Wu J., Zhou Y., Qiu Y., Zhang Z., Li X. (2022). Parametric Study of Spot Size and Multi-Elemental Quantification of Geomaterials under Complex Matrix Conditions Using Fiber-Optic Laser-Induced Breakdown Spectroscopy. Spectrochim. Acta.

[25] Zhang L., Ji S., Gu S., Huang X., Palmer J.E., Giewont W., Wang F.F., Tolbert L.M. (2020). Design Considerations for High-Voltage Insulated Gate Drive Power Supply for 10-KV SiC MOSFET Applied in Medium-Voltage Converter. IEEE Trans. Ind. Electron..

[26] Fortes F.J., Laserna J.J. (2010). The Development of Fieldable Laser-Induced Breakdown Spectrometer: No Limits on the Horizon. Spectrochim. Acta Part B At. Spectrosc..

[27] Dubey S., Kumar R., Rai A.K., Pati J.K., Kiefer J., Rai A.K. (2021). Rapid Analysis of Chemical Composition and Physical Properties of Gemstones Using LIBS and Chemometric Technique. Appl. Sci..

[28] Vrenegor J., Noll R., Sturm V. (2005). Investigation of Matrix Effects in Laser-Induced Breakdown Spectroscopy Plasmas of High-Alloy Steel for Matrix and Minor Elements. Spectrochim. Acta Part B At. Spectrosc..

[29] Rauschenbach I., Lazic V., Pavlov S.G., Hübers H.-W., Jessberger E.K. (2008). Laser Induced Breakdown Spectroscopy on Soils and Rocks: Influence of the Sample Temperature, Moisture and Roughness. Spectrochim. Acta Part B At. Spectrosc..

[30] Qiu Y., Wu J., Yu H., Gornushkin I.B., Li J., Wu Q., Zhang Z., Li X. (2020). Measurement of Trace Chromium on Structural Steel Surface from a Nuclear Power Plant Using Dual-Pulse Fiber-Optic Laser-Induced Breakdown Spectroscopy. Appl. Surf. Sci..

[31] Shaik N.B., Pedapati S.R., Taqvi S.A.A., Othman A.R., Dzubir F.A.A. (2020). A Feed-Forward Back Propagation Neural Network Approach to Predict the Life Condition of Crude Oil Pipeline. Processes.

[32] Lu S., Dong M., Huang J., Li W., Lu J., Li J. (2018). Estimation of the Aging Grade of T91 Steel by Laser-Induced Breakdown Spectroscopy Coupled with Support Vector Machines. Spectrochim. Acta Part B At. Spectrosc..

[33] Bakthavatchalam B., Shaik N.B., Hussain P.B. (2020). An Artificial Intelligence Approach to Predict the Thermophysical Properties of MWCNT Nanofluids. Processes.

[34] Sanjana K., Babu M.S., Sarathi R., Chillu N. (2022). Classification of Polluted Silicone Rubber Insulators by Using LIBS Assisted Machine Learning Techniques. IEEE Access.

[35] Bellou E., Gyftokostas N., Stefas D., Gazeli O., Couris S. (2020). Laser-Induced Breakdown Spectroscopy Assisted by Machine Learning for Olive Oils Classification: The Effect of the Experimental Parameters. Spectrochim. Acta Part B At. Spectrosc..

[36] Diaz D., Molina A., Hahn D.W. (2020). Laser-Induced Breakdown Spectroscopy and Principal Component Analysis for the Classification of Spectra from Gold-Bearing Ores. Appl. Spectrosc..

[37] Ipohorski M., Luppo M.I., Castillo-Guerra R., Ovejero-García J. (2003). Failure Analysis of a Steam Valve Stem. Mater. Charact..

[38] Liao F., Wang M., Tu L., Wang J., Lu L. (2019). Micromechanical Fracture Model Parameter Influencing Factor Study of Structural Steels and Welding Materials. Constr. Build. Mater..

[39] Balasubramanian V. (2008). Relationship between Base Metal Properties and Friction Stir Welding Process Parameters. Mater. Sci. Eng. A.

[40] Yu Z., Chen Y., Liu P., Wang W. (2015). Accelerated Simulation of Chloride Ingress into Concrete under Drying–Wetting Alternation Condition Chloride Environment. Constr. Build. Mater..

[41] Qiu Y., Wang A., Liu Y., Huang D., Wu J., Li J., Zhang Z., Li X., Wu Q. (2020). The Effect of Inter-Pulse Delay on the Spectral Emission and Expansion Dynamics of Plasma in Dual-Pulse Fiber-Optic Laser-Induced Breakdown Spectroscopy. Phys. Plasmas.

[42] Zeaiter M., Roger J.M., Bellon-Maurel V. (2006). Dynamic Orthogonal Projection. A New Method to Maintain the on-Line Robustness of Multivariate Calibrations. Application to NIR-Based Monitoring of Wine Fermentations. Chemom. Intell. Lab. Syst..

[43] García S., Fernández A., Luengo J., Herrera F. (2009). A Study of Statistical Techniques and Performance Measures for Genetics-Based Machine Learning: Accuracy and Interpretability. Soft Comput..

[44] El Haddad J., Canioni L., Bousquet B. (2014). Good Practices in LIBS Analysis: Review and Advices. Spectrochim. Acta Part B At. Spectrosc..

[45] Tao H., Wang P., Chen Y., Stojanovic V., Yang H. (2020). An Unsupervised Fault Diagnosis Method for Rolling Bearing Using STFT and Generative Neural Networks. J. Frankl. Inst..

[46] Begam S.S.J.V., Selvachandran G., Ngan T.T., Sharma R. (2020). Similarity Measure of Lattice Ordered Multi-Fuzzy Soft Sets Based on Set Theoretic Approach and Its Application in Decision Making. Mathematics.

[47] Saqlain M., Jafar N., Moin S., Saeed M., Broumi S. (2020). Single and Multi-Valued Neutrosophic Hypersoft Set and Tangent Similarity Measure of Single Valued Neutrosophic Hypersoft Sets. Neutrosophic Sets Syst..

[48] Mohebali B., Tahmassebi A., Meyer-Baese A., Gandomi A.H. (2020). Probabilistic Neural Networks: A Brief Overview of Theory, Implementation, and Application. Handb. Probabilistic Models.

[49] Alweshah M., Rababa L., Ryalat M.H., Al Momani A., Ababneh M.F. (2022). African Buffalo Algorithm: Training the Probabilistic Neural Network to Solve Classification Problems. J. King Saud Univ.-Comput. Inf. Sci..

[50] Chen G., Davies A.E., Banford H.M. (1999). Influence of Radiation Environments on Space Charge Formation in/Spl Gamma/-Irradiated LDPE. IEEE Trans. Dielectr. Electr. Insul..

[51] Qiu Y., Wu J., Zhang Z., Liu T., Xue F., Hang Y., Wu Y., Yu H., Li X. (2019). Comparisons of Laser-Produced Plasma in Atmosphere between Fiber-Delivery and Direct-Focusing Laser Pulse. Spectrochim. Acta Part B At. Spectrosc..

